# Supplementary material for: Discovery of plasma messenger RNA as novel biomarker for gastric cancer identified through bioinformatics analysis and clinical validation
Source: PeerJ. 2019 Jun 18;7:e7025. doi: 10.7717/peerj.7025 (PMC6587939; doi:10.7717/peerj.7025)
Supplement: Table S1 [file peerj-07-7025-s001.docx]

**Supplementary Table 1 Primer sequences**

| Primer | Sequence | Length of PCR product(bp) | Tm(ºC) |
| --- | --- | --- | --- |
| COL6A3 | tctgttcctctttgacggct  ccaccttgacatcatcgctg | 146 | 58 |
| SERPINH1 | ctgtccggtgcatcatcatg  gccatgttcttcaagccaca | 115 | 58 |
| PLEKHG1 | tgcacttaaaacccagcgac  accccagagttagccttcac | 187 | 58 |
| PGA4 | gaagcacaacctcaacccag  atgccgatagtgccgaagta | 117 | 58 |
| KCNE2 | atcctggtgagcactgtgaa  ttctcatggatggtggcctt | 140 | 58 |
| GIF | gctcaaatcctcccttccct  tgttagatgcagaggtgggg | 130 | 58 |
